# Combined effects of salinity and intermittent hypoxia on mitochondrial capacity and reactive oxygen species efflux in the Pacific oyster, *Crassostrea gigas*

**DOI:** 10.1242/jeb.246164

**Published:** 2023-08-03

**Authors:** Jennifer B. M. Steffen, Eugene P. Sokolov, Christian Bock, Inna M. Sokolova

**Affiliations:** ^1^ Department of Marine Biology, Institute of Biological Sciences, University of Rostock, 18059 Rostock, Germany; ^2^ Leibniz Institute for Baltic Research, Leibniz Science Campus Phosphorus Research Rostock, 18119 Warnemünde, Germany; ^3^ Integrative Ecophysiology, Alfred-Wegener-Institute Helmholtz Centre for Polar and Marine Research, 27570 Bremerhaven, Germany; ^4^ Department of Maritime Systems, Interdisciplinary Faculty, University of Rostock, 18059 Rostock, Germany

**Keywords:** Hypoxia tolerance, Mitochondria, Aerobic capacity, High-resolution respirometry, Oxidative stress marker, AmplexRed

## Abstract

Coastal environments commonly experience fluctuations in salinity and hypoxia–reoxygenation (H/R) stress that can negatively affect mitochondrial functions of marine organisms. Although intertidal bivalves are adapted to these conditions, the mechanisms that sustain mitochondrial integrity and function are not well understood. We determined the rates of respiration and reactive oxygen species (ROS) efflux in the mitochondria of oysters, *Crassostrea gigas*, acclimated to high (33 psu) or low (15 psu) salinity, and exposed to either normoxic conditions (control; 21% O_2_) or short-term hypoxia (24 h at <0.01% O_2_) and subsequent reoxygenation (1.5 h at 21% O_2_). Further, we exposed isolated mitochondria to anoxia *in vitro* to assess their ability to recover from acute (∼10 min) oxygen deficiency (<0.01% O_2_). Our results showed that mitochondria of oysters acclimated to high or low salinity did not show severe damage and dysfunction during H/R stress, consistent with the hypoxia tolerance of *C. gigas*. However, acclimation to low salinity led to improved mitochondrial performance and plasticity, indicating that 15 psu might be closer to the metabolic optimum of *C. gigas* than 33 psu. Thus, acclimation to low salinity increased mitochondrial oxidative phosphorylation rate and coupling efficiency and stimulated mitochondrial respiration after acute H/R stress. However, elevated ROS efflux in the mitochondria of low-salinity-acclimated oysters after acute H/R stress indicates a possible trade-off of higher respiration. The high plasticity and stress tolerance of *C. gigas* mitochondria may contribute to the success of this invasive species and facilitate its further expansion into brackish regions such as the Baltic Sea.

## Teaser

**Summary:** *Crassostrea gigas* shows higher mitochondrial plasticity during hypoxia–reoxygenation stress when acclimated to low salinity, contributing to its ecological and invasive success in varying environments.

## INTRODUCTION

Coastal environments are dynamic and highly productive ecosystems, but they are vulnerable to multiple natural and anthropogenic stressors. Sessile benthic organisms that inhabit these dynamic habitats must rely on physiological adaptations to cope with the challenges posed by these stressors (Grieshaber et al., 1994). Hypoxia, a severe oxygen deficiency that can last for hours, weeks or even months, is becoming increasingly prevalent in marine environments worldwide, particularly in coastal waters (Breitburg et al., 2018, 2019). Most marine animals rely on oxygen for mitochondrial metabolism and ATP production, making them highly susceptible to the negative effects of oxygen deficiency (Diaz and Rosenberg, 1995, 2008). Mitochondria are especially vulnerable to hypoxia-induced metabolic stress, and the degree of mitochondrial resilience to hypoxia generally corresponds to an organism's sensitivity to oxygen fluctuations. In hypoxia-intolerant species, hypoxia leads to severe damage to mitochondrial functions, depolarization of the mitochondrial membrane, and ATP deficiency, as well as Ca^2+^ overload (Piper et al., 2003; Solaini et al., 2010). While reoxygenation can restore ATP levels, it typically comes at the cost of the production of reactive oxygen species (ROS), which can cause damage to cellular macromolecules (Paradis et al., 2016). Hypoxia-intolerant organisms, including benthic invertebrates such as scallops, have been reported to suffer from oxidative damage, collapsed mitochondrial membrane potential, and a loss of oxidative phosphorylation (OXPHOS) capacity during hypoxia and reoxygenation (Ivanina and Sokolova, 2016; Ivanina et al., 2016). In contrast, some hypoxia-tolerant species, such as freshwater turtles of the genera *Chrysemys* and *Trachemys*, some fish species such as crucian carp, and intertidal bivalves, are able to preserve mitochondrial respiration, OXPHOS capacity and mitochondrial membrane potential during *in vivo* hypoxia–reoxygenation (H/R) stress (Galli and Richards, 2014; Pamenter, 2014; Sokolova, 2018; Sokolova et al., 2019). Moreover, in some hypoxia-tolerant species, severe oxygen deficiency can induce a transition to anaerobic ATP production, accompanied by the onset of metabolic rate depression that conserves energy and delays the onset of irreversible disruption of cellular homeostasis (Hochachka et al., 1996; Storey, 2002). In facultative anaerobes like marine bivalves, the transition between aerobic and anaerobic pathways of glucose oxidation is regulated at the phosphoenolpyruvate (PEP) branchpoint that plays an important role as a metabolic switch during oxygen deficiency. Pyruvate kinase (PK) and phosphoenolpyruvate carboxykinase (PEPCK) compete for a common substrate (PEP) directing it towards aerobic oxidation or anaerobic glycolysis, respectively (Bayne, 2017; Zammit and Newsholme, 1978). Changes in the PK/PEPCK activity ratio thus can indicate the relative activity of aerobic versus anaerobic metabolism and affect substrate provision to the mitochondria (Bayne, 2017; Ivanina et al., 2016; Zammit and Newsholme, 1978). Hence, gaining insights into the regulation of mitochondrial bioenergetics and redox balance can offer understanding of the mechanisms and constraints associated with the tolerance of benthic species towards oxygen fluctuations in coastal environments.

Besides fluctuating oxygen conditions, shallow habitats such as the Wadden Sea in the North Sea are characterized by fluctuating salinity (McLusky and Elliott, 2004). Salinity changes are usually caused by seasonal alterations in tidal cycle, precipitation, freshwater run off and evaporation. In times of anthropogenic change, altered frequencies of precipitation increase the pressure of salinity fluctuations (Durack et al., 2012). Fluctuating salinity induces shifts in osmotic balance that can negatively affect the cellular processes and integrity of organelles (Berger and Kharazova, 1997; Prosser, 1991). In molluscs, acclimation to different salinities requires regulation of intracellular osmolarity to match the ambient osmolarity and prevent excessive changes in cell volume (Evans, 2009; Shumway, 1977; Yancey, 2005). In the short term, marine bivalves seal their mantle cavity to prevent water–salt exchange with surrounding waters (Berger and Kharazova, 1997). This process relies on the ability of marine bivalves to survive the accumulation of metabolic end products in their tissues (Berger and Kharazova, 1997). In contrast, survival of long-term exposure to salinity stress requires the regulation of cell volume by organic and inorganic osmolytes to achieve the isosmotic state of the cellular environment relative to the habitat. This state is achieved by altered activity of *de novo* amino acid synthesis and protein breakdown (Meng et al., 2013; Zhao et al., 2012), and changes in the transport and retention of inorganic ions, particularly sodium (Podbielski et al., 2022).

The euryhaline Pacific oyster, *Crassostrea* (*Magallana*) *gigas* (taxonomic currently under debate, here the common name *C. gigas* is used), originally hailing from Pacific coastal areas and estuaries with a broad range of environmental conditions, has successfully invaded European waters since its introduction by humans in the mid-20th century (Sigwart et al., 2021; Troost, 2010). The species' success as an invasive organism can be attributed to its ability to tolerate diverse environmental factors. In response to H/R stress, *C. gigas* relies on mitochondrial function rearrangements to maintain energy homeostasis, which may contribute to its adaptability and stress tolerance (Sussarellu et al., 2013). A shifted proteome during H/R stress promotes mitochondrial resilience, leading to upregulation of the electron transport system (ETS) and suppression of pathways channelling electrons to ubiquinone, which may reduce ROS production (Sokolov et al., 2019). These findings suggest that maintenance of mitochondrial integrity and ATP synthesis capacity are crucial for oysters to tolerate H/R stress.

In osmoconformers such as oysters, metabolism is highly sensitive to changes in ambient salinity, which can result in shifts in cellular osmolarity and ion content (Berger and Kharazova, 1997). The resulting osmotic stress caused by ion disbalance and failing redox balance can have direct effects on metabolism in bivalves, leading to electron chain dysfunction, reduced coupling and high ROS efflux, thereby impairing mitochondrial efficiency and causing oxidative stress (Bal et al., 2022; Ballantyne and Moyes, 1987; Rivera-Ingraham et al., 2016a,b; Sokolov and Sokolova, 2019). Thus, high-salinity stress in *C. gigas* leads to respiratory disturbances, stimulates glycolysis, and leads to a depletion of glycogen reserves, indicating disruption of aerobic metabolism and energy deficiency (Chen et al., 2022; Fuhrmann et al., 2018). Conversely, low-salinity stress suppresses glycolysis and increases levels of proteins and energy reserves (carbohydrates and triglycerides) in oysters, indicating positive energy balance (Fuhrmann et al., 2018). These changes are modulated by AMP-dependent protein kinase (AMPK), a major regulator of cellular energy metabolism that directly affects mitochondria (Chen et al., 2022; Fuhrmann et al., 2018). These findings suggest that salinity stress may alter mitochondrial bioenergetics and redox balance, which may have implications for mitochondrial resilience towards other stressors such as H/R, warranting further investigation.

In this study, we investigated the impact of acclimation of different salinities on the resilience of mitochondria to intermittent hypoxia in the Pacific oyster, *C. gigas*, a stress-tolerant marine osmoconformer. Our hypothesis was that acclimation to low salinity would impair mitochondrial resilience to hypoxia and reoxygenation. To test this hypothesis, we acclimated oysters from the tidal habitat of the Wadden Sea to a low salinity of 15±1 psu, while another group was kept at their collection site salinity of 30±1 psu. After acclimation, the oysters were exposed to severe hypoxia for 24 h, followed by a recovery period of 1.5 h under normoxic conditions. To gain further insight into the intrinsic mechanisms of hypoxia tolerance and salinity acclimation, acute *in vitro* H/R exposure of isolated mitochondria was also conducted, as previously done in other studies (Adzigbli et al., 2022; Onukwufor et al., 2017; Sappal et al., 2015; Sokolov and Sokolova, 2019). Mitochondrial respiration, ROS efflux, activity of the PEP branchpoint enzymes and the levels of oxidative damage [malondialdehyde (MDA) protein conjugates and protein carbonyls (PC)] were measured to explore the impacts of acclimation to different salinities on the metabolic resilience to intermittent hypoxia in *C. gigas*.

**List of symbols and abbreviations**

ASW: artificial sea water
BSA: bovine serum albumin
ETS: electron transport system
FEL: fractional electron leak
H/R: hypoxia/reoxygenation
LEAK I: mitochondrial respiration respiring on substrates for respiratory complex I
LEAK I+II: mitochondrial respiration respiring on substrates for respiratory complex I and II
MDA: malondialdehyde
*Ṁ*_O_2__: oxygen consumption rate
OXPHOS: oxidative phosphorylation
OXPHOS CE: OXPHOS coupling efficiency
PBS: phosphate-buffered saline
PC: protein carbonyls
PEP: phosphoenolpyruvate
PEPCK: phosphoenolpyruvate carboxykinase
PK: pyruvate kinase
psu: practical salinity units
RCR: respiratory control ratio
ROS: reactive oxygen species
SUIT: substrate–uncoupler–inhibitor titration

## MATERIALS AND METHODS

### Chemicals

All chemicals were purchased from Carl Roth (Karslruhe, Germany), Sigma-Aldrich (Merck KGaA, Darmstadt, Germany) or Thermo Fisher Scientific (Waltham, MA, USA) unless otherwise noted and were of analytical grade or higher.

### Animal maintenance and experimental exposures

Adult oysters (mean±s.e.m. shell length: 124.76±14.52 mm) were obtained from the low intertidal zone of the German Wadden Sea near List/Sylt (55°01′42″N, 8°26′04″E) and transported within 48 h of collection to the University of Rostock, Germany, in coolers lined with seawater-soaked paper towels. For habituation to laboratory conditions, oysters were kept in recirculated temperature-controlled aquarium systems (Kunststoff-Spranger GmbH, Plauen, Germany) with aerated artificial seawater (ASW) (Tropic Marin^®^, Wartenberg, Germany) at a salinity of 33±1 psu and temperature of 15±0.5°C for 2 weeks. Salinity and temperature conditions were within the natural range of the oyster's habitat. Oysters were then randomly divided into two groups. One group was placed in a separate tank at 15±1°C and adjusted to a salinity of 15±1 psu at a rate of 2.5 psu per day. After the target salinity was achieved, oysters were transferred to a recirculated temperature-controlled aquarium system (Kunststoff-Spranger GmbH) with aerated ASW at a salinity of 15±1 psu and 15±1°C and acclimated for 3 months. The second (control) group was kept for the same duration at a salinity of 33±1 psu and 15±1°C. During the laboratory habituation and salinity acclimation, bivalves were fed *ad libitum* by continuous addition of a commercial live algal blend (DTs Premium Blend Live Marine Phytoplankton, Coralsands, Mainz Castel, Germany) according to the manufacturer's instructions (daily 80 ml per 500 l ASW) using an automated aquarium feeder.

For experimental hypoxia, bivalves were exposed to 24 h of severe hypoxia (<0.01% O_2_) by aeration of ASW with pure nitrogen (Westfalen AG, Münster, Germany) in air-tight glass jars (two oysters per 2 l ASW) at 15±0.5°C and the respective salinity. Oxygen concentration was monitored with an Intellical™ LDO101 Laboratory Luminescent/Optical Dissolved Oxygen (DO) Sensor (HACH, Loveland, CO, USA). During exposure, animals were not fed to prevent bacterial growth in the chambers. Subsequent to hypoxia exposure, a subset of animals was allowed to reoxygenate in normoxic ASW (21% O_2_) for 1.5 h. Incubation periods were chosen based on previous studies showing a strong physiological response within the first hours of reoxygenation (Falfushynska et al., 2020a; Haider et al., 2020). The control group was maintained in normoxia (21% O_2_) in recirculated temperature-controlled aquarium systems. Throughout experiments, no mortality was observed.

### Mitochondrial assays

Mitochondria were isolated from gills as described elsewhere (Ivanina and Sokolova, 2016; Kurochkin et al., 2011). Briefly, 1.1– 1.4 g of gill tissue were homogenized in ice-cold isolation buffer (pH 7.5, 760 mOsm;100 mmol l^−1^ sucrose, 200 mmol l^−1^ KCl, 100 mmol l^−1^ NaCl, 30 mmol l^−1^ Hepes, 8 mmol l^−1^ EGTA, 30 mmol l^−1^ taurine) in the presence of 1 mmol l^−1^ phenylmethylsulfonyl fluoride (PMSF), 2 µg ml^−1^ aprotinin and 2 mmol l^−1^ sodium orthovanadate using a Potter-Elvenhjem homogenizer at 200 rpm. The homogenate was centrifuged at 4°C, 2000 ***g*** for 8 min to remove cell debris and the supernatant was again centrifuged at 4°C, 8500 ***g*** for 8 min to isolate mitochondria. The pellet was resuspended in ice-cold assay buffer, the osmolarity of which corresponded to the respective salinity acclimation of oysters. Assay buffer for oysters acclimated to low salinity (525 mOsm, pH 7.5) contained 165 mmol l^−1^ sucrose, 50 mmol l^−1^ taurine, 10 mmol l^−1^ NaCl, 130 mmol l^−1^ KCl, 30 mmol l^−1^ Hepes, 10 mmol l^−1^ glucose, 1 mmol l^−1^ MgCl·6H_2_O, 10 mmol l^−1^ KH_­2_PO_4_ and 1% (w/v) bovine serum albumin (BSA), while assay buffer for oysters maintained at high salinity (750 mOsm, pH 7.5) consisted of 390 mmol l^−1^ sucrose, 50 mmol l^−1^ taurine, 10 mmol l^−1^ NaCl, 130 mmol l^−1^ KCl, 30 mmol l^−1^ Hepes,10 mmol l^−1^ glucose, 1 mmol l^−1^ MgCl·6H_2_O, 10 mmol l^−1^ KH_­2_PO_4_ and 1% (w/v) BSA.

Oxygen consumption and emission of ROS were measured in parallel by high-resolution respirometry and fluorometry using a 2k Oxygraph (Oroboros GmbH, Innsbruck, Austria). The oxygen electrodes were calibrated to 100% (by fully aerated assay medium) and 0% oxygen (by 30 mmol l^−1^ dithionite solution). ROS efflux was measured by the rate of emission of hydrogen peroxide (H_2_O_2_) by energized mitochondria as described elsewhere (Ouillon et al., 2021). Assay media contained 5 U ml^−1^ superoxide dismutase to convert superoxide radicals to detectable H_2_O_2_, 10 µmol l^−1^ AmplexRed as a reporter and 1 U ml^−1^ horseradish peroxidase (HRP) to catalyse the H_2_O_2_-dependent conversion of AmplexRed to its fluorescent form. Fluorometric sensors were calibrated with 0.2 µmol l^−1^ H_2_O_2_.

Mitochondrial oxygen consumption and H_2_O_2_ emission were measured using the following substrate–uncoupler–inhibitor titration (SUIT) protocol: 5 mmol l^−1^ pyruvate and 1 mmol l^−1^ malate to stimulate Complex I (LEAK I), 10 mmol l^−1^ succinate to stimulate Complex II (referred to hereafter as pre-*in vitro* anoxia LEAK I+II), 3.57 mmol l^−1^ ADP to achieve maximum ATP synthesis and OXPHOS activity and 10 µmol l^−1^ cytochrome *c* to assess mitochondrial integrity (referred to hereafter as pre-*in vitro* anoxia OXPHOS). We routinely use >10% increase in the OXPHOS respiration rate upon cytochrome *c* addition as a criterion to exclude the mitochondrial isolate due to a potentially poor quality. However, in the present study, no sample met this exclusion criterion so that all mitochondrial isolates were considered of sufficiently good quality for further analyses. To allow exposure to *in vitro* anoxia, mitochondrial suspensions were allowed to consume all oxygen in the chamber and maintained for 10 min at 0% O_2_. Subsequently chambers were reoxygenated and oxygen consumption and ROS efflux were recorded (referred to hereafter as post-*in vitro* anoxia recovery phase OXPHOS). Afterwards, the SUIT was continued with 5 µmol l^−1^ oligomycin to inhibit F_O_F_1_-ATPase (referred to hereafter as post-*in vitro* anoxia recovery phase LEAK I+II), stepwise titration (2.4 µmol l^−1^ steps) with carbonyl cyanide-chlorophenyl hydrazine (CCCP, final maximum concentration 14.3 µmol l^−1^) to uncouple ATP synthesis from ETS and thus measure maximum ETS activity, 9.5 µmol l^−1^ antimycin A to inhibit Complex III and 0.5 mmol l^−1^ *N*,*N*,*N*′,*N*′-tetramethyl-*p*-phenylenediamine (TMPD) and 2 mmol l^−1^ ascorbate to achieve maximum cytochrome *c* oxidase activity.

Protein concentrations of mitochondrial suspensions were measured using the Bradford assay (Thermo Fisher Scientific) and corrected for the BSA content of the media. Oxygen consumption and ROS efflux rates were standardized to mitochondrial protein content and expressed as nmol O_2_ min^−1^ mg^−1^ protein and nmol H_2_O_2_ min^−1^ mg^−1^ protein, respectively.

### Oxidative stress markers

Oxidative damage of mitochondria were assessed by determination of MDA–protein conjugates and protein carbonyls in mitochondrial suspensions from oysters acclimated to different salinities and oxygen regimes using indirect enzyme-linked immunosorbent assays (ELISA) (Ivanina and Sokolova, 2016; Matoo et al., 2013). Protein concentrations of 0.1 µg µl^−1^ for MDA ELISA and to 0.01 µg µl^−1^ for PC ELISA were obtained by diluting mitochondrial suspensions in phosphate-buffered saline (PBS). To prevent protein aggregation, dilutions were sonicated (Sonicator S-4000, Misonix, Famingdate, NY, USA; amplitude 24, 30 s). An MDA dilution series was prepared from a 1 mg ml^−1^ MDA–BSA–control standard (Cell Biolabs, San Diego, CA, USA) in 10 µg ml^−1^ fatty acid- and immunoglobulin-free BSA suspension. PC standards were prepared by oxidizing fatty acid- and immunoglobulin-free BSA with 30% H_2_O_2_ for 30 min. PC concentration of the oxidized BSA was determined spectrophotometrically as described elsewhere (Levine et al., 1990). Oxidized BSA standard was diluted to 10 µg ml^−1^ protein content with PBS and used to prepare the standard dilution series. ELISA plates were incubated with mitochondrial protein samples and standards at 4°C overnight and washed with PBS prior to further incubation.

For MDA, 1 mg ml^−1^ fatty acid- and immunoglobulin-free BSA was used to block plates for 2 h at 37°C prior to treatment with anti-MDA antibody (1:1000, ab27642, Abcam, Cambridge, UK). Subsequently, plates were incubated with anti-rabbit antibody conjugated with horseradish peroxidase (1:10,000, 111-035-003, Jackson ImmunoResearch Laboratories Inc., West Grove, PA, USA). For determination of PC, samples and standards were derivatized by incubation for 45 min with 5 mmol l^−1^ 2,4-dinitrophenylhydrazine (DNPH) in the dark prior to forming dinitrophenylhydrazone-protein carbonyl moiety (DNP). After washing plates with PBS–ethanol mixture (1:1 v/v), plates were blocked with 1 mg ml^−1^ fatty acid- and immunoglobulin-free BSA for 2 h at room temperature. Formation of DNP was assessed by incubation with anti-DNP antibody (1:1000, MAB2223, Merck Millipore, Burlington, MA, USA) followed by anti-mouse antibody (1:10,000, 115-035-03, Jackson Immuno Research Laboratories Inc.). Each antibody incubation was conducted for 1 h at room temperature. Bound antibodies were detected by the addition of horseradish peroxidase substrate TMB/E Ultra Sensitive (Merck Millipore) and 2 mol l^−1^ sulfuric acid to stop the reaction. Subsequently, development of colour was detected at 450 nm (SpectraMax iD3, Molecular Devices, LLC, San José, CA, USA).

### Enzyme activity

The activity of PK (EC 2.7.1.40) and PEPCK (EC 4.1.1.31) was spectrophotometrically measured in crude tissue extracts of hepatopancreas as described elsewhere (Simpfendörfer et al., 1995). Briefly, 200 mg tissue were homogenized in 10 mmol l^−1^ Tris-HCl (pH 7.0), 5 mmol l^−1^ Na_2_-EDTA, 0.1 mmol l^−1^ PMSF and 1 mmol l^−1^ dithiothreitol (DTT) using a FastPrep24 homogenizer with 6.5 m s^−1^ for 5 times 40 s (MP Biomedical, Santa Ana, CA, USA).

Assay media were as follows. PK: 50 mmol l^−1^ Tris-HCl (pH 7.0), 50 mmol l^−1^ KCl, 5 mmol l^−1^ MgSO_4_·7 H_2_O, 1 mmol l^−1^ ADP sodium salt, 0.2 mg ml^−1^ NADH, 5.5 U lactate dehydrogenase and 2.5 mmol l^−1^ PEP. PEPCK: 100 mmol l^−1^ Hepes (pH 7.0), 2.3 mmol l^−1^ MnCl_2_·4 H_2_O, 5 mg ml^−1^ KHCO_3_, 0.5 mmol l^−1^ IDP sodium salt, 0.2 mg ml^−1^ NADH, 10 U malate dehydrogenase and 15 mmol l^−1^ PEP. For both enzymes, reactions were started with the addition of PEP and monitored at 340 nm (SpectraMAx iD3, Molecular Devices, LLC). Absorbances was corrected by blank measurement. The activity of both enzymes was standardized to fresh tissue mass and expressed as nmol min^−1^ g^−1^ fresh tissue mass.

### Data analysis and statistics

Data were checked for normal distribution using the Shapiro–Wilk test in IBM^®^ SPSS^®^ Statistics (v. 25, IBM, Corp. Armonk, NY, USA) and for homogeneity of variances by the Brown–Forsythe test in Sigma Plot (v. 13.0.0.83, Systat Software Inc., San Jose, CA, USA). Outliers were excluded by box–whisker plots in IBM^®^ SPSS^®^ Statistics. In the case of non-normal distribution and/or non-homogeneity of variances, data were transformed by Box–Cox or Johnson transformation in Minitab (v.19, Minitab LLC., State College, PA, USA). Significant differences were tested by two-way ANOVA in SigmaPlot using *in vivo* oxygen and salinity regime as fixed factors. No significant factor interactions were detected by ANOVA for any of the studied parameters (*P*>0.05; data not shown). Therefore, the subsequent analyses focused on the effects of salinity and oxygen regime as determined by Tukey's honest significant differences (HSD) *post hoc* tests. Statistical comparison of *in vitro* oxygen treatments was analysed using paired *t*-test in Sigma Plot 13 within each salinity group, with *in vitro* oxygen treatment as a fixed factor. To analyse the *in vitro* data by paired *t*-test, we had to curate the data so that missing values in one of the groups (pre- or post-anoxia *in vitro*) were used as a criterion to remove that sample.

## RESULTS

### Effects of *in vivo* exposure of oysters to hypoxia and reoxygenation

#### Mitochondrial oxygen consumption

**Fig. 1.**
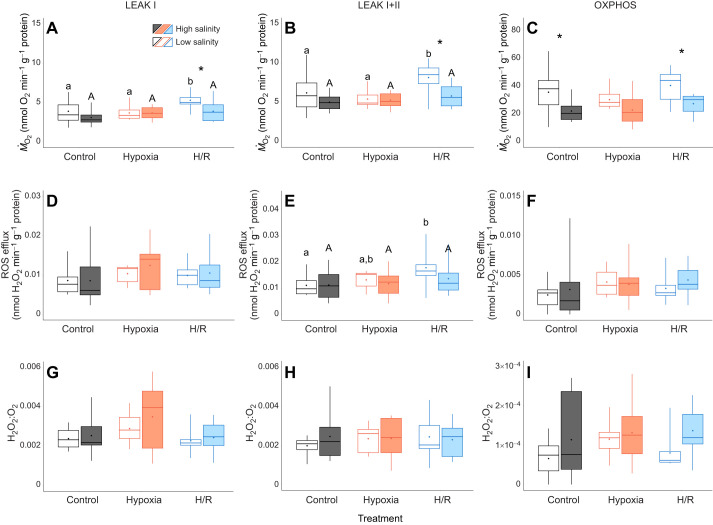
**Effect of salinity acclimation and short-term hypoxia–reoxygenation (H/R) stress on oxygen consumption, reactive oxygen species efflux and fractional electron leak of *Crassostrea gigas* mitochondria in resting (LEAK I and LEAK I+II) and active (OXPHOS) states.** Oxygen consumption (*Ṁ*_O_2__; A–C), reactive oxygen species (ROS) efflux (D–F) and fractional electron leak (FEL; H_2_O_2_:O_2_ ratio) (G–I) in resting mitochondria respiring on Complex I substrates pyruvate and malate (LEAK I) (left), in resting mitochondria respiring on complex I and II substrates pyruvate, malate and succinate (LEAK I+II) (middle) and active mitochondria (OXPHOS) (right). Experimental groups: control (21% O_2_); hypoxia, short-term (24 h) severe (<0.01% O_2_) hypoxia; H/R, short-term severe hypoxia (24 h at <0.01% O_2_) and subsequent 1.5 h reoxygenation (21% O_2_). Box–whisker plots: open, low-salinity (LS; 15 psu) acclimation; filled, high-salinity (HS; 33 psu) acclimation. Means are shown as points within corresponding box–whisker plots. Statistical significance (*P*<0.05) between oxygen treatments is indicated by different letters above plots and statistical differences (*P*<0.05) between salinity acclimation within one oxygen treatment are indicated by asterisks above plots. *N*=9–11, 8–10, 8–10, 8–10, 9–11 and 10–11 in LS control, HS control, LS hypoxia, HS hypoxia, LS H/R and HS H/R, respectively.

Acclimation to low salinity (15 psu) generally did not affect the baseline (LEAK) oxygen consumption rate (*Ṁ*_O_2__) of the oyster gill mitochondria (except during post-hypoxia recovery) but led to a ∼1.5-fold increase in the OXPHOS *Ṁ*_O_2__ compared with that of mitochondria from the high-salinity-acclimated oysters (Fig. 1A–C). In oysters acclimated to high salinity (33 psu), the baseline mitochondrial respiration with a Complex I substrate (LEAK I) or a combination of Complex I and II substrates (LEAK I+II) did not change during H/R stress compared with the normoxic control (Fig. 1A,B). In the oysters acclimated to low salinity (15 psu), exposure to hypoxia had no effect on the mitochondrial LEAK *Ṁ*_O_2__, but LEAK I and LEAK I+II *Ṁ*_O_2__ increased ∼1.4-fold during reoxygenation (Fig. 1A,B). Exposure to H/R stress showed no effect on OXPHOS *Ṁ*_O_2__ regardless of the acclimation salinity (Fig. 1C).

#### Mitochondrial ROS efflux and oxidative damage

ROS efflux rate and the fractional electron leak (FEL), calculated as the ratio of consumed O_2_ released as H_2_O_2_, were higher in the LEAK state than in the OXPHOS state mitochondria in all experimental groups (Fig. 1D–I). *In vivo* H/R stress and salinity acclimation did not alter H_2_O_2_ efflux from mitochondria in LEAK I and OXPHOS state (Fig. 1D,F). In the low-salinity-acclimated oysters, mitochondrial ROS efflux increased ∼1.6-fold during post-hypoxic recovery relative to the normoxic control in LEAK I+II state (Fig. 1E). However, FEL (H_2_O_2_:O_2_ ratio) remained unaltered by H/R stress and/or salinity acclimation (Fig. 1G–I).

**Fig. 2.**
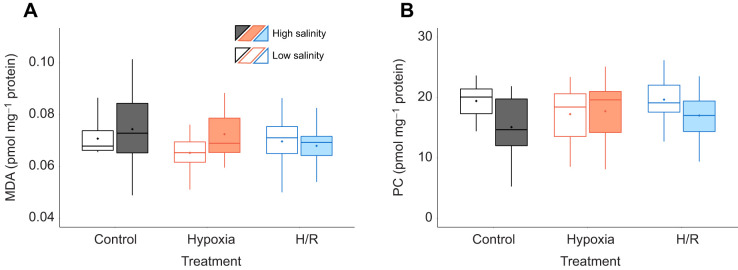
**Assessment of oxidative damage in mitochondrial proteins of oysters exposed to combined salinity and *in vivo* H/R stress.** (A) Malondialdehyde (MDA)–protein conjugates; (B) protein carbonyls (PC). Experimental groups: control (21% O_2_); hypoxia, short-term (24 h) severe (<0.01% O_2_) hypoxia; H/R, short-term severe hypoxia (24 h at <0.01% O_2_) and subsequent 1.5 h reoxygenation (21% O_2_). Box–whisker plots: open, low-salinity (LS; 15 psu) acclimation; filled, high-salinity (HS; 33 psu) acclimation. Means are shown as points within corresponding box–whisker plots. No significant differences between groups in these traits were found (*P*>0.05). *N*=10–11, 10, 10, 10, 10–11 and 10–11 in LS control, HS control, LS hypoxia, HS hypoxia, LS H/R and HS H/R, respectively.

Levels of the oxidative stress markers (MDA–protein conjugates and PCs) in oyster mitochondria remained unaltered by *in vivo* salinity acclimation and H/R stress (Fig. 2).

#### Mitochondrial coupling efficiency

**Fig. 3.**
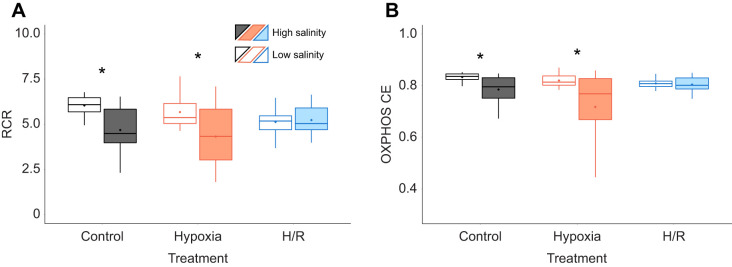
**Effect of salinity acclimation and short-term H/R stress on coupling efficiency of *C. gigas* mitochondria.** (A) Respiratory control ratio (RCR); (B) OXPHOS coupling efficiency [OXPHOS CE=1−(LEAK I+II/OXPHOS)]. Experimental groups: control (21% O_2_); hypoxia, short-term (24 h) severe (<0.01% O_2_) hypoxia; H/R, short-term severe hypoxia (24 h at <0.01% O_2_) and subsequent 1.5 h reoxygenation (21% O_2_). Box–whisker plots: open, low-salinity (LS; 15 psu) acclimation; filled, high-salinity (HS; 33 psu) acclimation. Statistical differences (*P*<0.05) between salinity acclimations are indicated by asterisks. *N*=10, 9–10, 10, 10, 9–10 and 10 in LS control, HS control, LS hypoxia, HS hypoxia, LS H/R and HS H/R, respectively.

Acclimation to low salinity led to a modest but statistically significant increase in the mitochondrial respiratory control ratio (RCR) and OXPHOS coupling efficiency (OXPHOS CE) in the oyster mitochondria compared with that of oysters from the high-salinity treatment (Fig. 3). H/R exposure showed no evidence of effect on the RCR and OXPHOS CE within each salinity acclimation group (Fig. 3). Similarly, exposure of isolated mitochondria to *in vitro* anoxia and reoxygenation showed no effect on RCR and OXPHOS CE regardless of the acclimation salinity (Fig. S1).

#### Activity of enzymes at the aerobic–anaerobic branch point

**Fig. 4.**
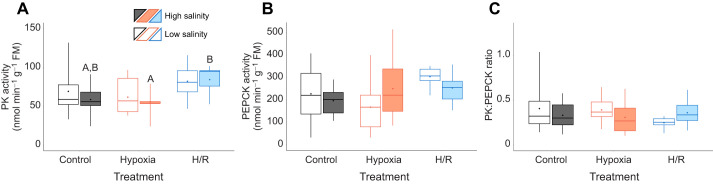
**Metabolic response of pyruvate kinase and phosphoenolpyruvate carboxykinase activity to combined salinity and H/R stress.** (A) Activity of pyruvate kinase (PK); (B) activity of phosphoenolpyruvate carboxykinase (PEPCK); (C) PK:PEPCK ratio. Experimental groups: control (21% O_2_); hypoxia, short-term (24 h) severe (<0.01% O_2_) hypoxia; H/R, short-term severe hypoxia (24 h at <0.01% O_2_) and subsequent 1.5 h reoxygenation (21% O_2_). Box–whisker plots: open, low-salinity (LS; 15 psu) acclimation; filled, high-salinity (HS; 33 psu) acclimation. Means are shown as points within corresponding box–whisker plots. Statistical significance (*P*<0.05) between oxygen treatments is represented by different letters above plots. *N*=11–12, 9–10, 9, 9–10, 8–11 and 9–11 in LS control, HS control, LS hypoxia, HS hypoxia, LS H/R and HS H/R, respectively.

In the high-salinity acclimation group, PK activity decreased in hypoxia and increased during reoxygenation so that the differences between the hypoxia-exposed and post-hypoxic recovery groups were significant (*P*<0.05) (Fig. 4A). In the low-salinity group, PK activity remained at the normoxic baseline levels during hypoxia and reoxygenation. PEPCK activity and PK:PEPCK ratios remained unchanged during H/R stress regardless of the acclimation salinity (Fig. 4B,C).

### Effects of acute *in vitro* exposure of isolated mitochondria to hypoxia and reoxygenation

#### Mitochondrial oxygen consumption and ROS efflux

**Fig. 5.**
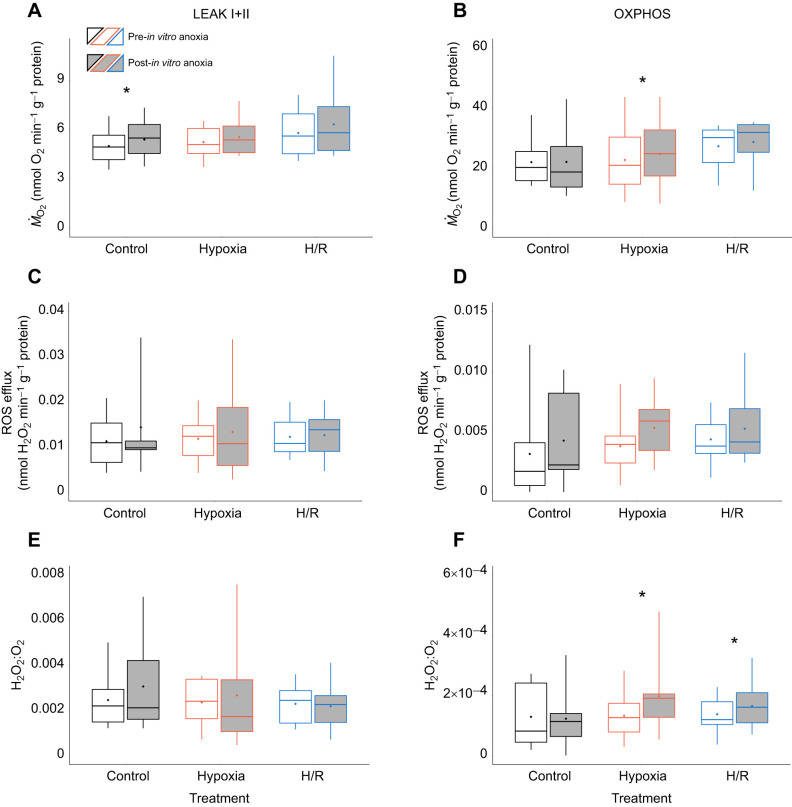
**Effect of *in vitro* H/R stress on oxygen consumption, ROS efflux and FEL of mitochondria in LEAK and OXPHOS states from oysters acclimated to high salinity.** Oxygen consumption (A,B), ROS efflux (C,D) and FEL (H_2_O_2_:O_2_ ratio; E,F) of mitochondria in LEAK I+II state (left) and OXPHOS state (right). Experimental groups: control (21% O_2_); hypoxia, short-term (24 h) severe (<0.01% O_2_) hypoxia; H/R, short-term severe hypoxia (24 h at <0.01% O_2_) and subsequent 1.5 h reoxygenation (21% O_2_). Box–whisker plots: open, pre-*in vitro* H/R stress; filled, post-*in vitro* H/R stress. Means are shown as points within corresponding box–whisker plots. Statistical differences (*P*<0.05) between pre- and post-*in vitro* H/R stress are indicated by asterisks above plots. Because of the focus on the effect of *in vitro* H/R stress, statistical results are only shown for paired *t*-tests. Data of open box–whisker plots are based on data of Fig. 1 curated for usage in paired *t*-tests as described in Materials and Methods. *N*=8–10, 8–10, 8–10, 8–10, 9–11 and 9–11 in control pre-anoxia, control post-anoxia, hypoxia pre-anoxia, hypoxia post-anoxia, H/R pre-anoxia and H/R post-anoxia, respectively.

**Fig. 6.**
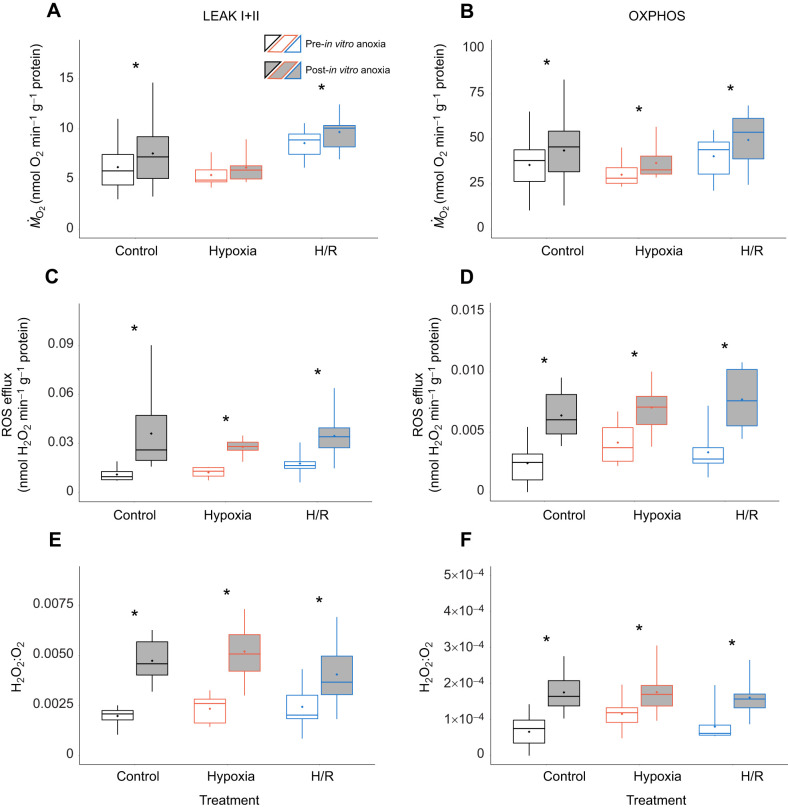
**Effect of *in vitro* H/R stress on oxygen consumption, ROS efflux and FEL of mitochondria in LEAK and OXPHOS states from oysters acclimated to low salinity.** Oxygen consumption (A,B), ROS efflux (C,D) and FEL (H_2_O_2_:O_2_ ratio; E,F) of mitochondria in LEAK I+II state (left) and OXPHOS state (right). Experimental groups: control (21% O_2_); hypoxia, short-term (24 h) severe (<0.01% O_2_) hypoxia; H/R, short-term severe hypoxia (24 h at <0.01% O_2_) and subsequent 1.5 h reoxygenation (21% O_2_). Box–whisker plots: open, pre-*in vitro* H/R stress; filled, post-*in vitro* H/R stress. Means are shown as points within corresponding box–whisker plots. Statistical differences (*P*<0.05) between pre- and post-*in vitro* H/R stress are indicated by asterisks. Because of the focus on the effect of *in vitro* H/R stress, statistical results are only shown for paired *t*-tests. Data of open box–whisker plots are based on data of Fig. 1 curated for usage in paired *t*-tests as described in Materials and Methods. *N*=9–11, 9–11, 8–10, 8–10, 9–11 and 9–11 in control pre-anoxia, control post-anoxia, hypoxia pre-anoxia, hypoxia post-anoxia, H/R pre-anoxia and H/R post-anoxia, respectively.

Generally, mitochondria from oysters acclimated to low salinity were more responsive to the acute *in vitro* anoxia that those from the high-salinity-acclimated oysters, regardless of the oxygen conditions (normoxia, hypoxia or post-hypoxic reoxygenation) under which the oysters were collected (Figs 5 and 6). In the high-salinity treatment group, there was a weak tendency for elevated LEAK and OXPHOS respiration of the isolated mitochondria after acute anoxia exposure *in vitro* (significant in the mitochondria isolated from the normoxic oysters for LEAK I+II and from the hypoxic oysters for OXPHOS) (Fig. 5A,B). Acute *in vitro* exposure to anoxia had no effect on the ROS efflux in mitochondria from the high-salinity-acclimated oysters (Fig. 5C,D). FEL in the LEAK state did not respond to acute *in vitro* anoxia exposure but increased in the OXPHOS state in the mitochondria isolated from hypoxic or recovering oysters in the high-salinity group (Fig. 5E,F). In the mitochondria of the low-salinity-acclimated oysters, LEAK and OXPHOS respiration, ROS efflux and FEL were stimulated by acute *in vitro* anoxia exposure of the mitochondria regardless of the oxygen conditions (normoxia, hypoxia or post-hypoxic reoxygenation) under which the oysters were collected (Fig. 6). Notably, a ∼1.2-fold increase in the oxygen consumption rates caused by the *in vitro* anoxic exposure was accompanied by ∼2-3-fold increase in the ROS efflux rates, leading to a noticeable increase in the FEL, particularly in the LEAK state (Fig. 6E,F).

## DISCUSSION

### Effects of salinity and H/R exposure on mitochondrial respiration and ROS efflux in oysters

Our study showed that salinity modulates the mitochondrial responses to long-term and acute hypoxia exposure in an euryhaline intertidal bivalve *C. gigas*. Interestingly, the mitochondrial performance of oysters was improved by acclimation to low (15 psu) salinity relative to the high (33 psu) salinity that corresponds to the salinity of the habitat where the oysters were collected. This was evident in the higher RCR and OXPHOS CE in the mitochondria of oysters from the low-salinity treatment, indicating improved mitochondrial coupling. Furthermore, mitochondria isolated from the gill of the low-salinity-acclimated oysters showed a higher plasticity during acute H/R stress, resulting in higher respiratory flux during post-hypoxic recovery compared with the mitochondria of high-salinity-acclimated oysters. Based on the mitochondrial coupling efficiency, a salinity of 15 psu might be considered closer to the optimum for the metabolic performance in the studied *C. gigas* population than a salinity of 33 psu. An earlier study using the same population of *C. gigas* acclimated to a salinity of 30 psu also showed better coupling, higher rates of ATP synthesis and ETS flux in the gill mitochondria measured at 450 mOsm (corresponding to a salinity of 15 psu) than in 900 mOsm (corresponding to a salinity of 30 psu) (Sokolov and Sokolova, 2019).

*Crassostrea gigas* was found to survive a wide window of salinity from 12 to 43 psu (Wiltshire, 2007) and can grow well at salinities above 15 psu (Calvo et al., 1999). Oysters are marine osmoconformers and thus are required to adjust their intracellular environment to the changing conditions of the surrounding seawater. Consequently, mitochondria need to be adapted to function across a wide range of osmolarities, depending on the habitat range. Mitochondria of euryhaline marine osmoconformers have broad osmotic tolerance as shown in the bivalves *Mercenaria mercenaria*, *Crassostrea virginica*, *Mya arenaria* and *Mytilus edulis* (Ballantyne and Storey, 1983; Ballantyne and Moyes, 1987; Haider et al., 2018; Noor et al., 2021)*.* Outside the osmotic tolerance range, mitochondria of euryhaline marine species show evidence of electron transport chain dysfunction, and high ROS production leading to oxidative stress (Bal et al., 2022; Paital and Chainy, 2012; Rivera-Ingraham et al., 2016a,b). Although the mitochondria of *C. gigas* showed improved performance at a salinity of 15 psu relative to 30 psu (Sokolov and Sokolova, 2019) or 33 psu (this study), no evidence of major ETS dysfunction or elevated ROS efflux was found at the higher acclimation salinity, consistent with notion of the broad salinity tolerance of this euryhaline species.

Mitochondria from *C. gigas* showed high resilience to prolonged (24 h) hypoxia and subsequent reoxygenation, regardless of the acclimation salinity. Respiration rates in the OXPHOS and LEAK state remained unchanged during H/R stress, except for upregulated respiration in post-hypoxic recovery predominantly under low salinity. Elevated mitochondrial oxygen consumption supports sufficient ATP synthesis capacity during reoxygenation. Evidence of a hypoxia-resilient mitochondrial phenotype maintaining high OXPHOS and ATP synthesis capacity during H/R stress was reported earlier in *C. gigas* (Sokolov et al., 2019; Steffen et al., 2020; Sussarellu et al., 2013) and other hypoxia-tolerant bivalves including *C. virginica* and *Arctica islandica* (Ivanina et al., 2012, 2016; Steffen et al., 2021). In contrast, hypoxia-intolerant bivalves such as scallops suffer from downregulation of OXPHOS caused by the loss of ETS capacity when exposed to hypoxia and reoxygenation (Ivanina et al., 2016). The ability to maintain high OXPHOS capacity and mitochondrial coupling during hypoxia and reoxygenation thus appears to be an adaptation to survive hypoxic periods in the intertidal zone (as in oysters and mussels) or anoxic sediments (as in *A. islandica*) not found in highly aerobic subtidal species such as scallops (Ivanina et al., 2012, 2016; Sokolov et al., 2019; Steffen et al., 2021; Sussarellu et al., 2013).

In oysters acclimated to low salinity, the post-hypoxic recovery process was associated with an upregulation of the respiration rate in LEAK state. Mitochondrial LEAK represents the rate of respiration required to counterbalance the depolarization of mitochondria caused by ion cycles that are not linked to ATP generation (Brand et al., 1994). Mildly elevated LEAK respiration is considered an important mitochondrial control mechanism to prevent excessive ROS formation (Brand, 1997; Miwa and Brand, 2003), whereas excessive LEAK rates might be associated with energy wastage leading to impaired OXPHOS efficiency and low coupling (Sokolova, 2023). In this study, we found that the mitochondria from oysters acclimated to low salinity were capable of maintaining normal RCR during post-hypoxic recovery despite the elevated proton leak rates with both Complex I and II substrates. This indicates that the observed modest stimulation of the proton leak during recovery does not result in suppressed mitochondrial efficiency. In contrast, the soft shell clam *M. arenaria* showed elevated LEAK respiration combined with lower OXPHOS respiration rates and a decrease in the OXPHOS coupling efficiency under the fluctuating oxygen regime (Ouillon et al., 2021). Both *C. gigas* and *M. arenaria* are stress-tolerant intertidal species with high invasive potential (Dutertre et al., 2009; Strasser, 1999), but the comparison of mitochondrial responses indicates that mitochondria of *C gigas* might be more resilient to oxygen fluctuations than those of *M. arenaria*.

Modest elevation of proton leak during post-hypoxic recovery might contribute to ROS control, as the FEL remained unchanged during post-hypoxic recovery in the mitochondria of *C. gigas*. Furthermore, no accumulation of oxidative lesions (MDA adducts or protein carbonyls) was found in the mitochondria of *C. gigas* exposed to H/R stress, regardless of the acclimation salinity, consistent with the notion of a resilient mitochondrial phenotype in this species. Hypoxia-tolerant bivalves commonly show stable levels of oxidative lesions during H/R stress compared with their hypoxia-intolerant counterparts (like scallops) (Ivanina and Sokolova, 2016; Ivanina et al., 2016) or hypoxia-intolerant vertebrate species (Cadenas, 2018; Honda et al., 2005; Zorov et al., 2014), which are prone to oxidative injury during H/R stress. This resistance to oxidative damage might be attributed to high levels of antioxidants and other cytoprotective molecules (including osmoprotectants and molecular chaperones) in stress-tolerant *Crassostrea* spp. (Sokolov et al., 2019; Sokolova et al., 2019; Steffen et al., 2020; Wei et al., 2022; Zhang et al., 2012)

### Metabolism at PEP branchpoint under variable oxygen and salinity regimes

In facultative anaerobes such as bivalves, breakdown products of glycolysis can be fed into the aerobic mitochondrial respiration (via pyruvate and acetyl-CoA) or anaerobic mitochondrial pathways of ATP production (via oxaloacetate) (Grieshaber et al., 1994; Ivanina et al., 2010, 2012). The PEP branchpoint is an important metabolic junction in the glucose oxidation of facultative anaerobes. PEP can be converted into pyruvate by the enzyme PK or into oxaloacetate by the enzyme PEPCK. The regulation of the PEP branchpoint is complex and is influenced by a variety of factors, including the availability of oxygen, intracellular pH and energy status, and depends on the PK/PEPCK activity ratio (Das et al., 2015; Saz, 1971; Zammit and Newsholme, 1978). Generally, high PK/PEPCK activity ratio leads to preferential channelling of PEP into aerobic respiration, and low PK/PEPCK activity ratio favours anaerobic formation of ATP, alanine and succinate (Bayne, 2017; Das et al., 2015; Simpfendörfer et al., 1995). In the present study, PK/PEPCK activity ratio in oyster gills remained unchanged during the H/R cycle. This indicates a lack of metabolic reorganization at the PEP branchpoint and might be due to the relatively short hypoxic exposure (24 h) used in our present study. Although *C. gigas* has been shown to transcriptionally regulate expression of *pk* and *pepck* within the first 6–12 h of hypoxic exposure (Sussarellu et al., 2012), most bivalves including oysters, scallops and clams do not rely heavily on anaerobic metabolism during short-term hypoxia (David et al., 2005; Greenway and Storey, 1999; Ivanina et al., 2010, 2016; Kurochkin et al., 2009). However, in sustained hypoxia, oysters do rely more heavily on anaerobic succinate-producing pathways of ATP formation indicated by the switch of PK and PEPCK activities (Bayne, 2017; Grieshaber et al., 1994). The green-lipped mussel *Perna viridis*, in contrast, exhibits a different response, with upregulation of PEPCK and elevated production of succinate occurring after only 24 h of hypoxia (Nusetti et al., 2010). Thus, the duration of hypoxic exposure appears to play a crucial role in determining the energy supply pathways utilized by these organisms.

### Intrinsic mitochondrial response mechanisms to acute anoxia and reoxygenation *in vitro*

Mitochondria are organelles with semi-autonomous capabilities, possessing their own DNA, protein translation machinery and quality control systems, which allow them to respond directly to H/R transitions independently of the rest of the cell (McBride et al., 2006). Previous research has shown that mitochondria in bivalve species, such as oysters and mussels, have intrinsic mechanisms of metabolic plasticity that involve functional changes in the ETS activity and reorganization of the mitochondrial proteome, regardless of the cellular environment (Adzigbli et al., 2022; Sokolov et al., 2019). When exposed to acute *in vitro* anoxia, isolated mitochondria from oysters and mussels respond by upregulating OXPHOS and modifying multiple proteins involved in ETS, tricarboxylic acid cycle, fatty acid and amino acid metabolism, and protein quality control (Adzigbli et al., 2022; Sokolov et al., 2019). Our study on *C*. *gigas* revealed that the response of mitochondria to acute short-term anoxia is influenced by acclimation to different salinities, but not by H/R exposure of the entire animal. Specifically, mitochondria from oysters acclimated to high salinity showed minimal changes in oxygen consumption or ROS efflux during acute H/R stress. In contrast, mitochondria from oysters acclimated to low salinity exhibited a strong upregulation of the OXPHOS and LEAK respiration rates after acute anoxia exposure *in vitro*. These findings suggest that acclimation to high salinity reduces the intrinsic mitochondrial plasticity to acute anoxia stress, which is observable in the mitochondria of low-salinity-acclimated oysters. Intrinsic plasticity of isolated mitochondria in response to various stimuli depends on mechanisms such as post-translational protein modifications (Falfushynska et al., 2020b; Mathers and Staples, 2019; Pagliarini and Dixon, 2006; Sokolov et al., 2021; Yang and Gibson, 2019), allosteric regulation of enzyme activity (Acin-Perez et al., 2011; Beauvoit and Rigoulet, 2001; Cherkasov et al., 2007) and proton-motive force-dependent regulation of respiration and ROS production (Brand, 2000; Jastroch et al., 2010; Lambert and Brand, 2004). In our present study, the exact mechanisms responsible for the differences in the intrinsic mitochondrial plasticity between the mitochondria of oysters acclimated to high and low salinity could not be determined requiring further investigation. Furthermore, previous studies on *C. gigas* mitochondria have also shown that lower osmolarity in the mitochondrial environment promotes faster ATP synthesis, higher ETS capacity and improved mitochondrial coupling (Sokolov and Sokolova, 2019). The increase in OXPHOS activity and ATP synthesis capacity in low-salinity-acclimated oysters after acute anoxia exposure may accelerate the restoration of their energy balance compared with high-salinity-acclimated oysters. However, this rapid recovery may come at the expense of redox disturbances, as indicated by higher rates of ROS efflux and elevated FEL rates in the mitochondria of low-salinity-acclimated oysters after acute anoxia stress. Similar findings have been observed in *Danio rerio*, a hypoxia-tolerant zebrafish species, where *in vitro* anoxia increased OXPHOS respiration rates but also ROS efflux (Napolitano et al., 2019). This suggests that the upregulation of oxygen consumption during post-hypoxic recovery may impose costs on mitochondrial functions, if ROS production exceeds the mitochondrial capacity to mitigate redox stress.

It is important to note that ROS play a dual role in cellular responses to H/R, not only causing harmful effects on cellular macromolecules but also acting as signalling molecules that regulate adaptive cellular responses to oxygen fluctuation through the activation of hypoxia-inducible factors and Nrf2-dependent transcriptional regulators (Sies and Jones, 2020; Sies et al., 2022; Zorov et al., 2014). The unexpected increase in ROS levels observed in oyster mitochondria exposed to low salinity and acute anoxia *in vitro* may therefore be linked to adaptive signalling through ROS to activate stress response pathways (Forman et al., 2010; Sokolova, 2018). Additional research is required to elucidate whether the increased release of ROS triggered by acute anoxia stress serves as a beneficial signalling mechanism or represents a trade-off between ATP production and redox balance in oyster mitochondria.

*In vivo* exposure of oysters to H/R stress did not affect the *in vitro* response of mitochondria during post-hypoxic recovery, regardless of salinity acclimation. However, long-term *in vivo* hypoxia exposure in sablefish resulted in mitochondria becoming more resilient to acute *in vitro* H/R stress, potentially related to reduced ROS production through increased proton conductance via higher LEAK respiration (Gerber et al., 2019). It is possible that the short-term hypoxic exposure in our study was not severe enough to activate adaptive plasticity of oyster mitochondria. Previous studies on vertebrates and invertebrates have found that robust maintenance or even enhancement of OXPHOS and ETS capacity during *in vivo* or *in situ* H/R exposure is a common trait in hypoxia-tolerant species, whereas hypoxia-intolerant species often suffer from loss of OXPHOS and ETS capacity (Ivanina et al., 2016; Paradis et al., 2016; Venditti et al., 2001). Oyster mitochondria displayed strong resilience to hypoxia in our study, which may be associated with a phenotype commonly observed in hypoxia-tolerant bivalve species characterized by unchanged or elevated OXPHOS capacity, ROS mitigation and prevention of ETS collapse during post-hypoxic recovery, partially independent of cellular mechanisms.

### Conclusions and outlook

Acclimation to low salinity appears to improve the mitochondrial performance of *C. gigas*, as indicated by higher RCR and better mitochondrial coupling. Oysters acclimated to low salinity also showed higher oxygen consumption during post-hypoxic recovery, indicating higher mitochondrial plasticity in response to H/R stress compared with that of those from high salinity. These results suggest that a salinity of 15 psu might be closer to *C. gigas*’ metabolic optimum, even though the studied population originated from a high-salinity (33 psu) habitat. Previous studies have also indicated better mitochondrial performance in low osmolarity for oysters (Sokolov and Sokolova, 2019). However, both high- and low-salinity oysters showed unchanged OXPHOS and LEAK respiration during H/R stress, demonstrating their high resilience to intermittent hypoxia (Ivanina et al., 2016; Sokolov et al., 2019; Sussarellu et al., 2013). Despite better mitochondrial performance in low salinity, high salinity did not cause severe mitochondrial damage or dysfunction during H/R stress, reflecting the broad salinity tolerance of *C. gigas* (Troost, 2010). However, salinity exposure affected the intrinsic response of isolated mitochondria to acute anoxia. Acclimation to low salinity permitted strong upregulation of respiration of mitochondria during post-hypoxic recovery, which may support the rapid reinstatement of cellular energy homeostasis. This stronger metabolic plasticity, however, came with a higher cost of mitochondrial maintenance, due to elevated ROS production. The characteristic features of a hypoxia-tolerant mitochondrial phenotype (such as elevated OXPHOS capacity and ROS mitigation during H/R stress) can thus be modulated by environmental salinity in euryhaline osmoconformers such as oysters. Mitochondrial performance typically correlates with organismal performance and fitness in ectotherms (Koch et al., 2021). Based on the mitochondrial performance, the studied invasive population of *C. gigas* has the metabolic capacity to perform well in low-salinity habitats, thus potentially spreading further into European brackish waters (Schmidt et al., 2008), provided the salinity barrier for larval recruitment is overcome (Ewers-Saucedo et al., 2020). Whether this high mitochondrial plasticity is indeed one of the adaptive traits of larvae and adult *C. gigas* allowing for the successful invasion of the Baltic Sea requires further investigation.

## Supplementary Material

10.1242/jexbio.246164_sup1 Supplementary information
