# Supplementary material for: Combined effects of salinity and intermittent hypoxia on mitochondrial capacity and reactive oxygen species efflux in the Pacific oyster, Crassostrea gigas
Source: J Exp Biol. 2023 Aug 3;226(15):jeb246164. doi: 10.1242/jeb.246164 (PMC10445735; doi:10.1242/jeb.246164)
Supplement: Supplementary information [file jexbio-226-246164-s1.pdf]

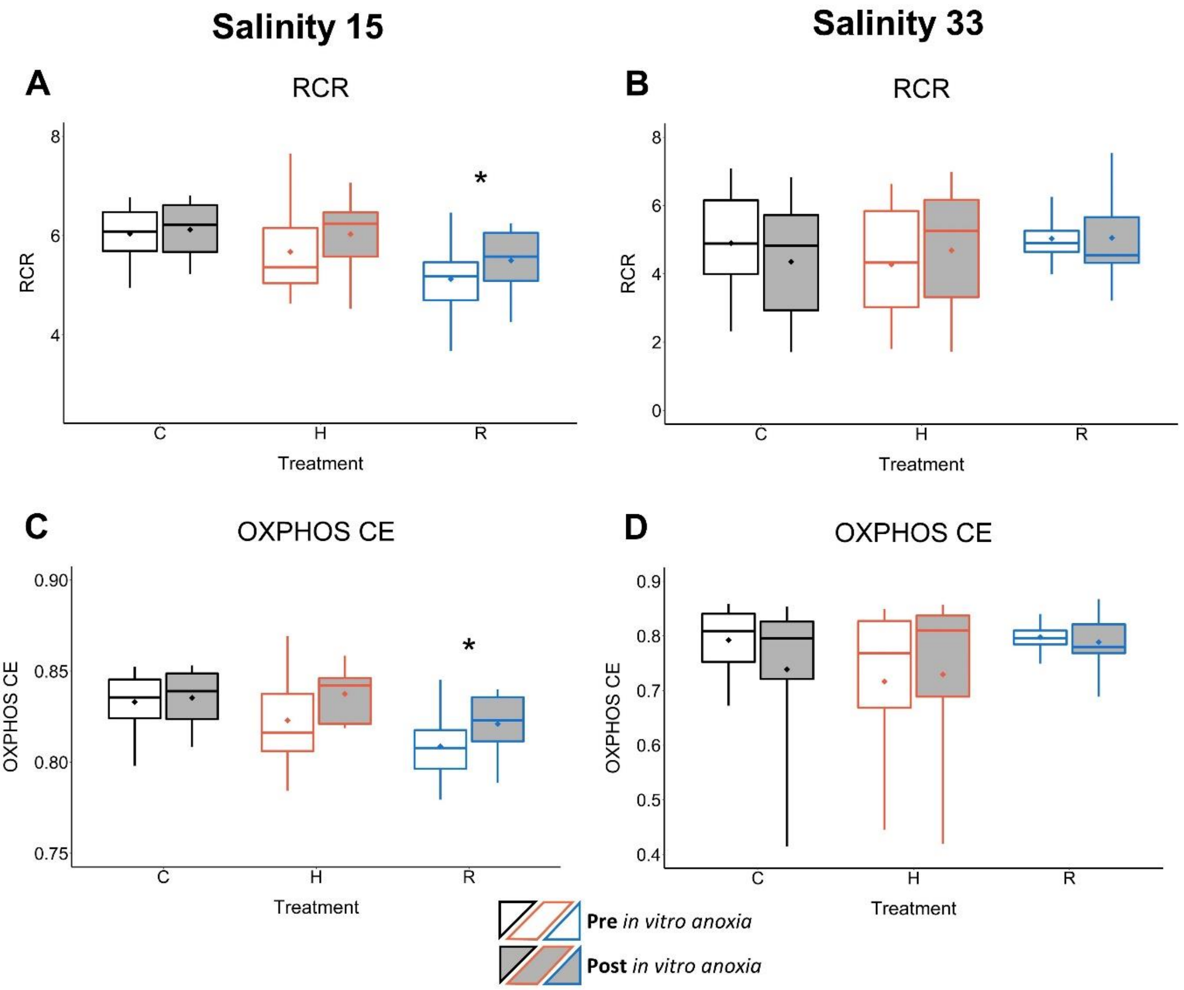

Fig. S1. Effect of *in vitro* H/R stress on coupling efficiency of *C. gigas* mitochondria from oysters exposed to combined *in vivo* salinity and H/R stress.

(A, B) Respiratory control ratio (RCR); (C,D) OXPHOS coupling efficiency (OXPHOS CE); (A,C) mitochondria from oysters acclimated to salinity 15; (B,D) mitochondria from oysters acclimated to salinity 33. Experimental groups: C, control (21% O2); H, short-term (24h) severe (<0.01% O2) hypoxia; R, short-term severe hypoxia (24 h at <0.01% O2) and subsequent 1.5 h reoxygenation (21% O2). Empty Box-Whisker plot – pre in vitro H/R stress; grey-filled Box-Whisker plot – post in vitro H/R stress. Means are shown as points within corresponding BOX-Whisker plots. Statistical differences (P <0.05) between pre and post in vitro H/R stress are represented by asterisk (*) above plots. Plots not marked with a star are not significantly different (P > 0.05). Due to the focus on the effect of in vitro H/R stress, statistical results are only shown for paired t-test. Data of empty Box-Whisker plots are based on data of Fig.2 curated for usage in paired t-tests as described in the Material and Method section. N of LS = 10, 10, 9-10, 9-10, 9-10 and 9-10 in C pre anoxia, C post anoxia, H pre anoxia, H post anoxia, R pre anoxia and R post anoxia, respectively. N of HS = 9-10, 9-10, 10, 10, 10 and 10 in C pre anoxia, C post anoxia, H pre anoxia, H post anoxia, R pre anoxia and R post anoxia, respectively.

Journal of Experimental Biology • Supplementary information
